# Transcriptomic and Proteomic Analyses Reveal the Potential Mode of Action of Chondrocyte Sheets in Hyaline Cartilage Regeneration

**DOI:** 10.3390/ijms21010149

**Published:** 2019-12-24

**Authors:** Eriko Toyoda, Masato Sato, Takumi Takahashi, Miki Maehara, Eri Okada, Shiho Wasai, Hiroshi Iijima, Ken Nonaka, Yuka Kawaguchi, Masahiko Watanabe

**Affiliations:** 1 Department of Orthopaedic Surgery, Surgical Science, Tokai University School of Medicine, 143 Shimokasuya, Isehara, Kanagawa 259-1193, Japan; 2 DNA Chip Research Inc., 1-15-1, Minato-ku, Tokyo 105-0022, Japan; 3 CellSeed Inc., 2-5-10, Aomi, Koto-ku, Tokyo 135-0064, Japan

**Keywords:** cartilage regeneration, cell sheet, xenogeneic transplantation model, efficacy

## Abstract

Chondrocyte sheet transplantation is a novel and promising approach to treating patients who have cartilage defects associated with osteoarthritis. Hyaline cartilage regeneration by autologous chondrocyte sheets has already been demonstrated in clinical research. In this study, the efficacy of polydactyly-derived chondrocyte sheets (PD sheets) as an allogeneic alternative to standard chondrocyte sheets was examined using an orthotopic xenogeneic transplantation model. In addition, the expression of genes and the secreted proteins in the PD sheets was analyzed using a microarray and a DNA aptamer array. The efficacy of PD sheets with respect to cartilage defects was assessed using histological scores, after which the expressions of genes and proteins exhibiting a correlation to efficacy were identified. Enrichment analysis of efficacy-correlated genes and proteins showed that they were associated with extracellular matrices, skeletal development, and angiogenesis. Eight genes (ESM1, GREM1, SERPINA3, DKK1, MIA, NTN4, FABP3, and PDGFA) exhibited a positive correlation with the efficacy of PD sheets, and three genes (RARRES2, APOE, and PGF) showed a negative correlation for both transcriptomic and proteomic analyses. Among these, MIA, DKK1, and GREM1 involved in skeletal development pathways and ESM1 involved in the angiogenesis pathway exhibited a correlation between the amount of secretion and efficacy. These results suggest that these secreted factors may prove useful for predicting PD sheet efficacy and may therefore contribute to hyaline cartilage regeneration via PD sheets.

## 1. Introduction

It is well known that articular cartilage does not regenerate easily. Spontaneous repair of cartilage defects cannot be expected following damage by trauma or osteoarthritis (OA). In fact, OA is designated as a degenerative cartilage disease, with about half of those over 40 years old being estimated as potential patients [1]. Although OA accounts for the majority of cartilage injuries that require treatment, there is no established definitive therapy for OA.

Chondrocyte sheets can be created without using material such as animal-derived collagen by using temperature-responsive culture devices such as thermo-responsive polymer grafted culture dishes [2,3], where cells can adhere, proliferate, and form into a sheet. The sheet can be harvested without enzymatic digestion by lowering the temperature. By using this technology, chondrocyte sheets can be collected and triple-layered and then transplanted onto cartilage lesions while retaining the extracellular matrix and adhesion molecules that are produced by the chondrocytes themselves [4]. Because chondrocyte sheets can adhere to the recipient site without sutures, the chondrocytes will remain at the site of implantation.

Chondrocyte sheet transplantation is therefore a promising approach to treating articular cartilage lesions. Articular cartilage regeneration with hyaline cartilage, which is important for joint function, has been demonstrated using partial-thickness and osteochondral defect models of rats, rabbits, and pigs [4,5,6,7,8].

Treatment with chondrocyte sheets using the patient’s own cells has been performed on 20- to 60-year-old patients who had knee joint cartilage damage caused by traumatic or OA degeneration. The articular cartilage was repaired to hyaline cartilage, and the treatment improved the clinical joint condition score [9]. These results suggest that cell sheet transplantation could become the definitive treatment for OA-associated articular cartilage lesions.

Based on years of experience with allograft transplantation, it is well known that cartilage tissue is immune tolerant. Allogeneic cartilage fragments are commercially available and have been widely transplanted in the United States [10]. It is therefore considered that the use of allogeneic chondrocyte sheets can be an applicable treatment. In considering the application of allogeneic chondrocyte sheets to the treatment of joint disease, we have investigated the application of chondrocytes obtained from the surgical remains of juvenile polydactyly patients. To date, we have reported that polydactyly-derived chondrocytes have excellent proliferative capacities that allow them to establish a layered structure and to form sheets that have similar characteristics to autologous chondrocyte sheets, such as the expression of mesenchymal cell surface markers and the production of various cartilage anabolic factors [11].

Because most polydactyly surgery is performed in infancy, highly proliferative chondrocytes can be obtained. If the number of chondrocytes obtained from a single donor is insufficient to fabricate many sheets, the chondrocytes are culture expanded and cryopreserved as a material for future polydactyly-derived chondrocyte sheet (PD sheet) fabrication.

However, the cartilaginous tissues obtained from polydactyly surgery include the parts that may differentiate into secondary ossification center, growth plate, articular cartilage, or bone. It is also well known that chondrocytes are easily dedifferentiated on expansion culture on dishes, and we can assume that the PD sheet characteristics will be affected by variations in the culture period and passage number. For these reasons, it is necessary to clarify the effects of the diversity associated with allogeneic cell sheets on the effectiveness of cell sheet transplantation.

We have previously reported that the efficacy of human adult knee chondrocyte sheets on osteochondral defects can be directly evaluated via a xenogeneic transplantation model involving immunosuppressant-treated rabbits [12]. In this study, we evaluated the efficacy of PD sheets for osteochondral defects in terms of this xenogeneic model, with gene expression and the secreted proteins of transplanted PD sheets also being evaluated. Transcriptomic and proteomic analyses revealed the gene expression profiles and production factors that are characteristic of effective or less effective PD sheets. In addition, the biological functions of the molecules that contributed to PD sheet effectiveness were estimated.

## 2. Results

### 2.1. Effect of PD Sheets on Hyaline Cartilage Regeneration

**Figure 1:**
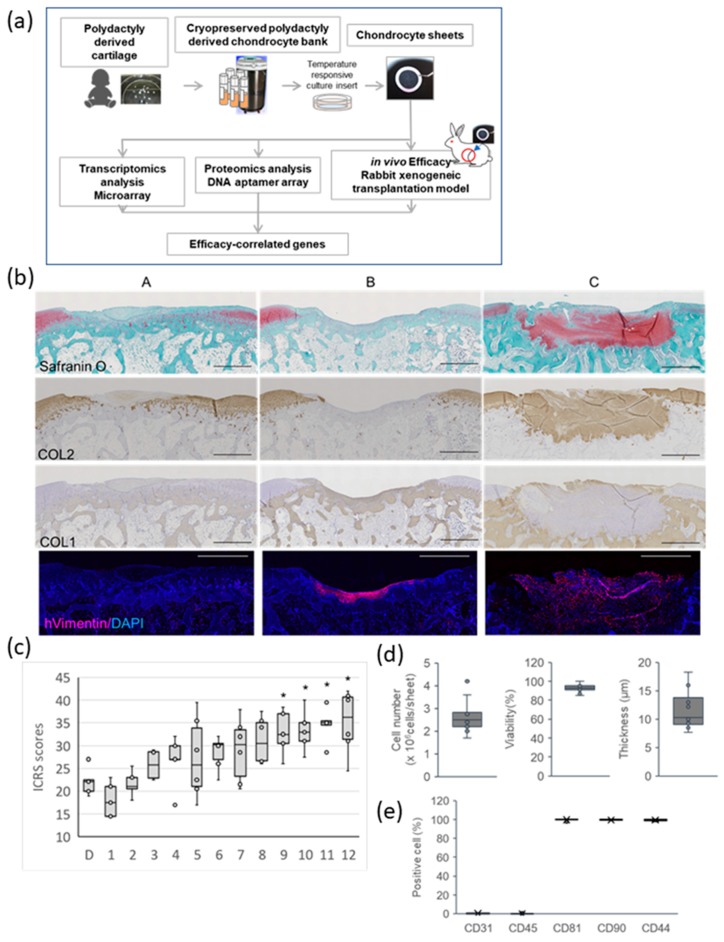
Comparison of 12 polydactyly-derived (PD) chondrocyte sheets with respect to their efficacy on hyaline cartilage regeneration in a rabbit xenogeneic transplantation model: (**a**) Overview of the study. (**b**) Representative images, using histological staining with safranin-O and immunohistochemical staining of COL2, COL1, and vimentin for rabbit knee cartilage defects with and without treatment with cell sheet transplantation. A: no sheet transplantation, B: poor cartilage repair (#1 in (**c**)), C: excellent cartilage repair (#12 in (**c**)). Scale bar = 1 mm. (**c**) ICRS scores for the PD sheets: Each circle indicates the average ICRS score for rabbits in a group. D represents a group without PD sheet transplantation. The number indicates each lot of PD sheets in Supplementary Table S1. * *p* < 0.05. (**d**) Distribution of PD sheet characteristics: Cell sheets were dispersed by enzymatic digestion, with cell numbers and the viability of cell suspension being determined. (**e**) PD sheets showing similar surface marker expressions.

Our overall experimental design is shown in Figure 1a. To assess the impact of diversity of PD sheets on the effectiveness and transcriptomic and proteomic profiles, PD sheets were created for 12 lots of PD chondrocytes obtained from 10 donors. These lots involved different passage numbers, expansion under different culture conditions, and different cryopreservation media (see Supplementary Table S1). Figure 1b,c shows the results of the PD sheet evaluation for osteochondral defects in the rabbit orthotopic xenograft model. Column A in Figure 1b shows representative results of the no-treatment control group. Osteochondral defects were mainly filled by bone-like and fibroblastic cartilage-like tissue that stained for type I collagen (COL1) and absent for type II collagen (COL2). Columns B and C are representative results of PD sheet transplantation groups of different lots that had the lowest and highest International Cartilage Repair Society (ICRS) scores, indicating poor cartilage repair and excellent cartilage repair, respectively. Column B showed incomplete filling with fibrous cartilage. Column C exhibited hyaline cartilage regeneration in which the tissue clearly stained for safranin-O, indicating proteoglycan deposition, and for COL2, indicating hyaline cartilage regeneration at 4 weeks after transplantation. These results indicated the replacement of the original osteochondral defect area by hyaline cartilage at this time point (4 weeks). We expect the eventual replacement of the hyaline cartilage by subchondral bone in the areas which are typically filled with bone in healthy cartilage at longer periods (12 weeks) as we have observed in a previous study [6]. The ICRS score at four weeks in the untreated group, indicated as D, was 22.13 ± 3.09 (*n* = 5), and ICRS scores for the transplantation groups indicated as #1 to #12 varied between 18.1 and 35.2 (see Figure 1c). Among PD sheet transplantation groups, four lots demonstrated statistically significant efficacy in repairing osteochondral defects and several lots clearly failed in doing so.

In both column B and C, the presence of human vimentin positive cells in regenerated tissue was observed. These results suggest that the regenerated tissue was derived from the transplanted PD sheet in both cases.

Figure 1d shows the property distributions for the PD sheets with respect to thickness, cell number, and viability of the cells contained in the PD sheets. The PD sheets formed piled-cell structures, and their thicknesses were 7.7–18.3 μm (average 11.52 ± 3.23 μm). Cell numbers also varied across the range 1.7–4.2 × 10^6^ cells per PD sheet (average 2.65 ± 0.67 × 10^6^). The PD sheets were digested enzymatically, and their surface marker expressions were analyzed. Flow cytometric analysis of the dispersed cells showed that the markers that are generally expressed by blood lineage cells, CD31 (platelet endothelial cell adhesion molecule-1), and CD45 (leukocyte common antigen) were almost always negative. For CD81 (cluster of differentiation 81) and Mesenchymal stem cell (MSC) markers, CD90 (also known as Thy-1) and CD44 (a receptor for hyaluronic acid) were more than 95% positive for all lots. We have reported that these markers are expressed in both adult chondrocyte sheets, which exhibited effectiveness in a previous clinical study and PD sheets [10]. There was almost no significant difference in the expression of these cell surface markers between the lots (see Figure 1e).

### 2.2. Gene Expression Profiles Associated with the Effectiveness of PD Sheets

**Figure 2:**
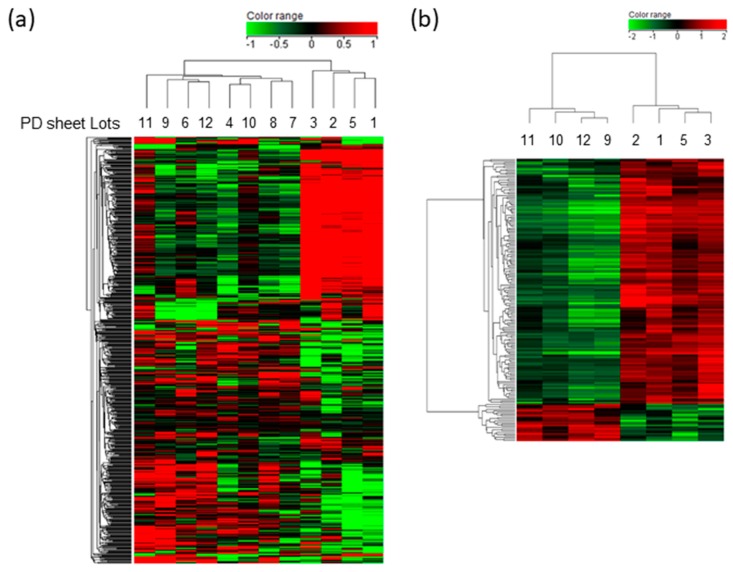
Heat map and hierarchical clustering of efficacy-correlated gene expressions: PD sheets exhibited a distinct gene expression profile according to their in vivo efficacy. Red indicates high expression levels, and green represents low expression levels. (**a**) Analyzed using efficacy correlated 443 gene set. (**b**) Differentially expressed 171 genes in effective and less effective lots of PD sheets.

To identify transcriptomic characteristics related to PD sheet effectiveness, the gene expression of the 12 lots of PD sheets, which had been examined with respect to their efficacy via the orthotopic xenograft model, was analyzed using microarrays. We extracted genes based on the correlation between the array signals and the ICRS scores, obtaining 205 genes showing a positive correlation with the ICRS score and 238 genes showing a negative correlation. The genes for the 20 highest positive and negative correlations are listed in Supplementary Tables S2 and S3, respectively. Figure 2a shows the results of a cluster analysis of 443 extracted genes via a heat map of gene expression. We can consider these as candidate marker genes for efficacy-related characteristics. For the five lots of PD sheets with the lowest ICRS scores, four lots are classified into the same cluster, suggesting that gene groups that contribute to this clustering may be an index to exclude those that are less effective. We further identified 171 genes with changes in expression of greater than 2-fold between the effective 4 lots that exhibited efficacy in Figure 1c and less effective lots that fall into the cluster. In the less effective lots, 149 genes were upregulated and 22 genes were downregulated (absolute fold change > 2; adjusted *p*-value < 0.05). Figure 2b shows clear separation of PD lot groups, suggesting that the gene set could be candidate markers that identify less effective PD sheets.

### 2.3. Function Prediction by Efficacy-Related Genes

**Figure 3:**
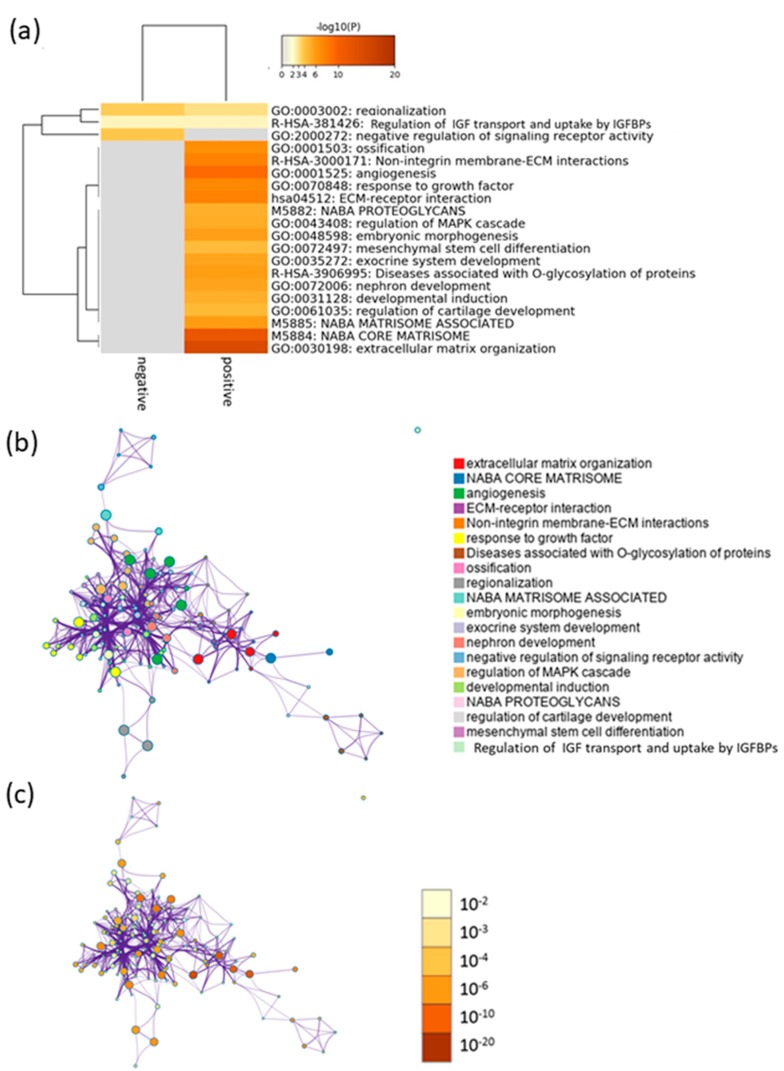
Meta-enrichment analysis of the efficacy-associated genes: (**a**) Heat map of the top 20 enriched clusters based on positive and negative correlation gene lists. Colors represent statistical significance, with gray indicating a lack of significance. ECM: extracellular matrix, IGF: insulin-like growth factor, IGFBPs: insulin-like growth factor binding proteins. (**b**) Enrichment network visualization: Cluster annotations are shown color-coded. The size of the node indicates the number of genes included in the ontology terms or pathways. (**c**) The color scale for statistical significance was applied to the same cluster network.

To select the marker genes that have reasonable biological relevance for efficacy, we clarified the biological functions of efficacy-related gene by performing an enrichment analysis of 443 genes using Metascape [13]. The results suggest that the positively correlated genes were involved in cartilage development and mesenchymal stem cell differentiation (Figure 3). In addition, some of the genes were involved in angiogenesis and extracellular matrix organization.

**Figure 4:**
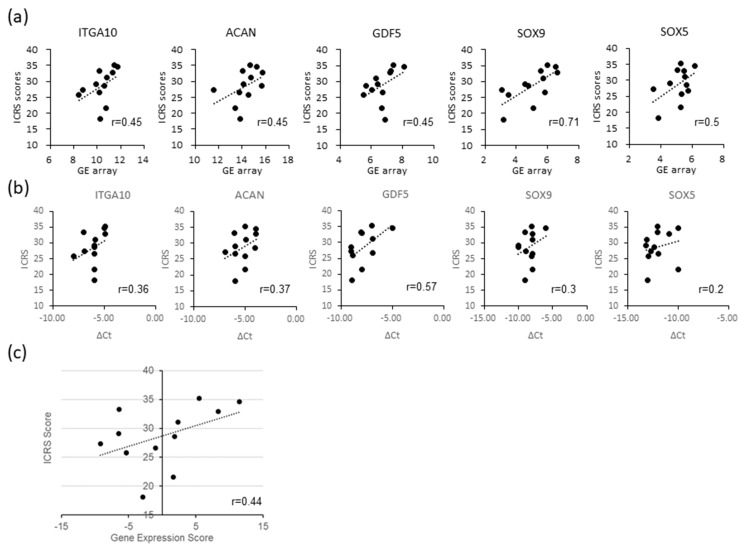
Potential efficacy-related markers for PD sheets: (**a**)The scatterplots of selected genes show a correlation between normalized microarray signal and ICRS scores of PD sheets (*n* = 12). (**b**) A weak correlation between gene expression in PD sheets that is consistent with (**a**) and their ICRS scores were validated by RT-PCR. The horizontal axis shows the gene expression level in terms of ΔCt values that were calculated by subtracting the Ct value of target genes from the Ct value of actin beta (ACTB) as a reference gene. (**c**) Scatterplot of gene expression score (GES) calculated from gene expression levels of marker genes.

Table 1 shows the efficacy-related genes described and annotated in terms of enriched gene ontology (GO). Some genes associated with GO:0030198 extracellular matrix organization were also associated with GO:0061035 regulation of cartilage development. We selected five GO-annotated genes in Figure 4a for reverse transcription polymerase chain reaction (RT-PCR) assay. The expressions of aggrecan (ACAN), integrin alpha 10 (ITGA10), growth differentiation factor 5 (GDF5), SRY-box transcription factor 9 (SOX9), and SRY-box transcription factor 5 (SOX5) were validated via RT-PCR, and the correlation with the ICRS score was analyzed. The results show that there was a correlation between the ΔCt with respect to actin beta (ACTB) and the ICRS scores for ACAN, ITGA10, GDF5, and SOX9 but not SOX5 (Figure 4b). ACAN, ITGA10, GDF5, and SOX9 were selected as the efficacy-related marker components. After Z-score normalization, the gene expression score (GES) was calculated by each predictive marker gene based on weighted linear functions (GES = (ΔCt (ACAN) × 3 + ΔCt (ITGA10) × 2 + ΔCt (GDF5) × 0.4 + ΔCt (SOX9) × 1). The correlation coefficient of these data sets was 0.44 for ICRS scores (Figure 4c).

### 2.4. Analysis of Secretory Factors Related to PD Sheet Effectiveness

**Figure 5:**
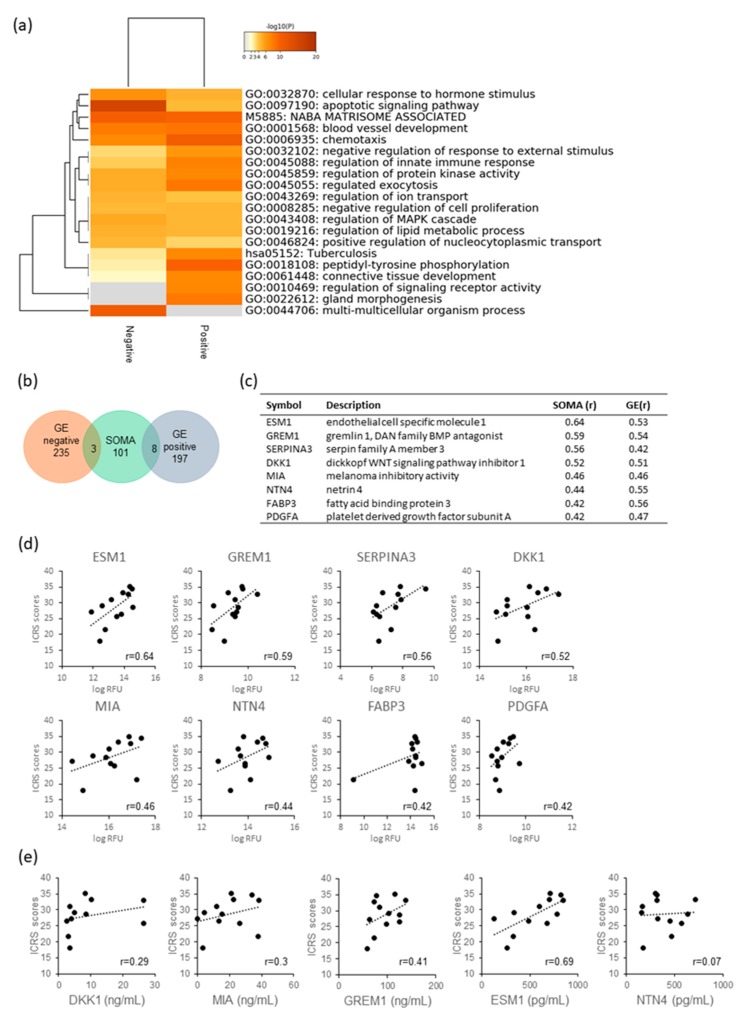
Analysis of proteins secreted by PD sheets: (**a**) Meta-enrichment analysis of selected proteins. (**b**) Venn diagram of genes selected from the microarray and SOMA scan assay. (**c**) List of genes exhibiting positive correlation in both mRNA expression (GE) and protein secretion (SOMA). (**d**) Scatterplots between ICRS score and relative protein amount for supernatants of the PD sheets as examined by SOMAscan (log10 relative fluorescence unit: logRFU). (**e**) Scatterplots between ICRS score and protein concentration as determined by ELISA.

It might be expected that the humoral factors continuously supplied from a PD sheet contribute to the promotion of hyaline cartilage regeneration. Therefore, we analyzed the relative amounts of secreted proteins from PD sheets using the slow off-rate modified aptamers (SOMA) scan platform [14,15] and their correlation with ICRS scores. Among the protein factors that can be analyzed with SOMAscan, 49 proteins having a positive correlation with efficacy score and 63 proteins having a negative correlation were extracted. The factors for the 20 highest positive and negative correlations are listed. (See Supplementary Tables S4 and S5.) Figure 5a shows the results of the enrichment analysis of the efficacy-correlated factors. Negatively correlated factors were concentrated in the GO:apoptotic signaling pathway. Factors that showed a positive correlation are enriched in connective tissue development, which is the parent term of cartilage development, and blood vessel development, which is the parent term of angiogenesis. These pathways were similar to those for enrichment in gene expression analysis. Among the proteins that showed an efficacy-related expression of genes, eight proteins were identified in the supernatant that correlated with effectiveness: ESM1, GREM1, SERPINA3, DKK1, MIA, NTN4, FABP3, and PDGFA (Figure 5b–d). Three proteins showed a negative correlation: retinoic acid receptor responder 2 (RARRES2), apolipoprotein E (APOE), and placental growth factor (PGF).

Of these, we focused on DKK1, which is expected to demonstrate OA-preventing effects [16]; GREM1, known to be involved in chondrocyte differentiation [17]; MIA, which is a cartilage differentiation marker and anabolic factor [18,19,20]; and NTN4 and ESM1, which are both angiogenesis related factors. The amounts of these factors were quantified via enzyme-linked immunosorbent assay (ELISA), and the results showed that the amount of protein varied between PD sheets, with the ranges being 2.6 to 26.6 ng/mL for DKK1, 58.2 to 138 ng/mL for GREM1, 0 to 38.4 ng/mL for MIA, 153 to 714 pg/mL for NTN4, and 128 to 849 pg/mL for ESM1. Among them, NTN4 failed to exhibit correlation with ICRS scores in ELISA. For the concentration of MIA and DKK1, a weak correlation with ICRS scores was observed. For GREM1 and ESM1, a moderate correlation was demonstrated (Figure 5e).

## 3. Discussion

In this study, we have confirmed that using PD sheets is effective in the orthotopic xenograft model, similar to using adult knee chondrocyte sheets. In the fabrication of human adult chondrocyte sheets, chondrocytes proliferate in the 2D-culture environment, causing the expression of Col2A1 to be reduced and the chondrocyte sheets to be considered dedifferentiated. However, it has been found that they redifferentiate into hyaline cartilage in the transplanted joint for both animal model [12] and human clinical research [9]. Using PD sheets, we have found hyaline cartilage-like tissue in defect areas derived from human cells, indicating that PD sheets also redifferentiate to hyaline cartilage in vivo.

The results of using an in vivo imaging system to track chondrocyte-sheet orthotopic transplants in rats indicate that most of the luciferase signal of the allogeneic chondrocytes in the joint will disappear within a few months [21], suggesting that allograft-derived tissue is replaced by recipient-derived tissue in the long term. Our results suggest that, at least in the short term, PD sheets may act as cartilage progenitor cells.

It has been suggested that the chondrocyte sheets that adhere to the cartilage lesion act as a barrier against catalytic factors while prevent the outflow of cartilage-tissue matrices [4,22]. Moreover, there is an implication that anabolic factors produced from chondrocyte sheets act as paracrine factors for promoting cartilage regeneration [11,23,24]. Our study shows that the expression of genes and the secretion of protein factors associated with the extracellular matrix, cartilage development, ossification, and angiogenesis are both involved in the efficacy of PD sheets. We previously reported that PD sheets secrete MIA as an anabolic factor [11], and it is confirmed again in this study. In addition, we have identified other candidates for anabolic factors (GREM1, DKK1, and PDGF) that contribute to the efficacy of PD sheets.

GREM1 and DKK1, of which secretion was confirmed by PD sheets and exhibited a correlation to efficacy, are known to be involved in Wnt signal regulation, and Wnt signal disruption in articular cartilage is thought to cause cartilage homeostasis failure [25,26,27,28]. They are reported to suppress hypertrophic chondrocyte differentiation and to increase cartilage surface and quiescent chondrocyte layers [17]. Suppression of the onset of OA in DKK1-transgenic mice has also been reported [16]. PDGF-AA is indicated as a cartilage trophic factor that promotes chondrocyte proteoglycan production and chondrocyte proliferation [29,30] and a decrease in PDGF-AA promotes cartilage degeneration [31].

Our results suggest that PD sheets promote the regeneration of hyaline cartilage by producing these factors. Because PD sheets are living tissues, they may be able to tune their factor production during the process of redifferentiating into hyaline cartilage according to the microenvironment: the cartilage surface layer, the deeper layers, and the subchondral bone. Production of these factors may be a useful marker for predicting the effectiveness of the PD sheets when selecting donors for cell sheet production or improving culture methods.

In addition, our study shows that the production of NTN4 and ESM1, which have not been reported as having any specific role in cartilage tissue, and ESM1 was correlated with the efficacy of PD sheets. NTN4 is a netrin family factor that has been reported to promote osteoblast differentiation [32] and to modify SMAD signaling in chondrocytes [33], suggesting that NTN4 may have some effect on the chondrocytes themselves and the subchondral bone. NTN4 has also been reported to suppress angiogenesis [34,35]. ESM1 is a dermatan sulfate proteoglycan of which the expression is enhanced by vascular endothelial growth factor (VEGF) [36,37]. It has been reported that ESM1 expresses higher in the deep cartilage layer than in other parts of the cartilage [38], implying a biological role related to chondrocyte differentiation. Rocha et al. report that ESM1 increases the bioavailability of VEGF-165 by competitively binding to fibronectin [39]. Because PD sheets express abundant fibronectin, it is assumed that the ESM1 secreted from the PD sheets binds and saturates the fibronectin in the PD sheets themselves or in the recipient tissue. ESM1 may prevent binding of VEGF and invagination of new blood vessels.

Angiogenesis in articular cartilage is thought to cause cartilage degeneration and osteophyte formation. We previously reported that osteophyte formation is suppressed by anti-VEGF antibody administration in an anterior-cruciate-ligament transection rabbit model [40,41]. These results suggest that the suppression of angiogenesis may be a promising approach to OA prevention. The NTN4 and ESM1 produced by PD sheets may contribute to the regeneration of hyaline cartilage by suppressing angiogenesis and subsequent calcification during the repair of a cartilage defect.

SERPINA3 is a member of the serine protease family and has been reported as a gene of which the expression level changes when MSC differentiate into chondrocytes [42]. FABP3 is a fatty acid binding protein which is required for the accumulation of intracellular lipid droplets and has been reported to increase MSC survival [43,44]. However, because FABP3 is a protein that is normally localized in the Golgi body, the increased amount in the supernatant may be related to the cell death of cells expressing FABP3. The contribution of these factors to effectiveness is currently unknown, and further analysis is required.

One limitation of our study was that the results were obtained from a xenograft model using immunosuppressants. There could therefore be some differences in the engraftment and rejection processes between xenogeneic and allogeneic transplantation, which should be considered in human applications. The engraftment of PD sheets is considered to be very important for effectiveness, and some factors could promote engraftment in xenogeneic transplantation rather than promote the regeneration of cartilage.

In regenerative medicine using allogeneic cells, which may well vary in their properties because of donor differences, stabilizing the properties of the tissue engineering products is an inevitable issue for clinical use. To address this issue, it is essential to identify biological characteristics that contribute to in vivo efficacy. This would then enable validation of donor cells and culture conditions based on identified biological characteristics.

PD sheets are transplanted to histoincompatibile patients in parallel with microfracture treatment in our ongoing clinical study, so the contact between bone marrow and PD sheets should be considered. The potential immune modulatory function of PD sheets may affect their efficacy. We have demonstrated the efficacy of chondrocyte sheet transplantation in allogeneic settings in rats, rabbits, and minipigs without immunosuppression [5,6,7,8]. Additionally, many allogeneic osteochondral transplantations in histoincompatibile patients have been conducted safely [10,45,46]. However, the influence of histoincompatiblility on efficacy of PD sheet transplantation is still unknown, as we are currently evaluating the immune response in the PD sheet transplant recipients. To reduce the immune response through the contact between blood and PD sheets, PD sheet transplantation without microfracture or its application in early OA patients could be considered, as chondrocyte sheets have demonstrated their effectiveness on partial thickness defects of articular cartilage [4,8].

In this study, we have identified biological pathways and associated genes that may contribute to hyaline cartilage regeneration by PD sheet transplantation. Although further improvements are needed, we have proposed gene expression scores and relevant secreted factors that could be used as markers to predict the in vivo efficacy of PD sheets. To use these markers for efficacy prediction, the correlation with efficacy needs to be further analyzed via independent data and could be replaced by better genes and the thresholds for the markers need to be determined. Eventually, the contribution of these factors to the efficacy of PD sheets will need to be confirmed in human clinical studies before they can be used as quality markers.

## 4. Materials and Methods

### 4.1. Ethics Statement

The experiments were performed under the approval and guidance of the Clinical Research Review Committee of Tokai University School of Medicine. Informed consent was obtained from the parents or guardians of the donors in all cases. Some surgical specimens were irreversibly de-identified. All experiments handling human cells and tissues were performed in line with the tenets of the Declaration of Helsinki.

The animal experiments were approved by the Institutional Animal Experiment Committee at Tokai University and were performed in accordance with the guidelines of the Institutional Regulations for Animal Experiments and the Fundamental Guidelines for Proper Conduct of Animal Experiments and Related Activities in Academic Research Institutions under the jurisdiction of the Japanese Ministry of Education, Culture, Sports, Science, and Technology for animal handling and care.

### 4.2. Fabrication of PD Sheets

#### 4.2.1. Preparation of Chondrocytes

Chondrocytes were isolated according to the preparation procedure described previously [11]. Briefly, cartilaginous tissue obtained from juvenile polydactyly patients (11 patients, aged 8–23 months, five girls, four boys, and two de-identified donors) was digested with 5 mg/mL CLS1 (Worthington Biochemical Corp., Lakewood, NJ, USA) in a preparation medium (DMEM/F12, Gibco, Waltham, MA, USA) supplemented with 20% fetal bovine serum and 1% antibiotic–antimycotic solution (Gibco) for 2–3 h at 37 °C in a humidified atmosphere of 5% CO_2_ and 95% air. The isolated chondrocytes were washed with the preparation medium and seeded on culture dishes. After the cells adhered and started to proliferate, the medium was replaced with a culture medium (the preparation medium with 100 μg/mL ascorbic acid (Nissin Pharmaceutical, Yamagata, Japan)). In two cases, tissues were minced into pieces of 1 mm^3^ or less, placed on culture dishes in the preparation medium, and cultured until the cells became subconfluent. Chondrocytes were harvested using TrypLE Express (Thermo Fisher Scientific, Tokyo, Japan) and cryopreserved either with STEM-CELLBANKER™ (ZENOAQ, Fukushima, Japan) or TC-Protector cell freezing media (KAC Co., Ltd., Kyoto, Japan). Some donors were passaged up to five times before being cryopreserved.

#### 4.2.2. Fabrication of PD Sheets

Twelve lots of cryopreserved chondrocytes were thawed, passaged once in the culture medium, seeded on temperature-responsive culture inserts (CellSeed Inc., Tokyo, Japan) at 1 × 10^4^ cells/cm^2^, and cultured for two weeks. The PD sheets were divided into four groups for use in efficacy evaluation, evaluation of characteristics, analysis of gene expression, and analysis of protein secretion.

#### 4.2.3. Evaluation of Characteristics of PD Sheets

To determine the cell number, PD sheets were digested with TrypLE Express (Thermo Fisher Scientific) for 30 min at 37 °C followed by incubation with 0.25 mg/mL Collagenase-P (Roche, Basel, Switzerland) for 30 min at 37 °C. The dispersed cells were washed with culture medium, and cell number and viability were determined by trypan blue exclusion assay. To determine the thickness of the PD sheets, each PD sheet was embedded in Tissue-Tek O.C.T. Compound (Sakura Finetech, Tokyo, Japan) and frozen at −80 °C. Cross sections of the PD sheet at 10 μm thicknesses were cut and mounted onto glass slides, air-dried, and fixed with 4% paraformaldehyde in 0.01 M phosphate buffer for 30 min at RT. The sections were stained with H&E according to standard protocols. Microscopic images were captured, and the distances between pairs of points were measured using a BZ-8000 microscope (Keyence, Osaka, Japan). For the flow cytometry, dispersed cells were suspended in Ca/Mg-free phosphate buffer saline (PBS) containing 0.2% human serum albumin (Sigma-Aldrich, Tokyo, Japan) and 1 mM ethylenediaminetetraacetic acid (EDTA: Wako Chemical Co. Ltd., Tokyo, Japan). They were then incubated with five antibodies: CD31/FITC (clone: 5.6E) and CD45/FITC (clone: J.33) were supplied by Beckman Coulter; CD81/APC (clone: JS-81), CD90/APC (clone: 5E10), and CD44/FITC (clone: G44-26) were supplied by BD Biosciences (San Diego, CA, USA). The stained cells were washed once and analyzed using a FACSVerse flow cytometer (BD Biosciences).

### 4.3. Evaluation of the Efficacy of PD Sheets for Osteochondral Defects

#### 4.3.1. Immunosuppressed Rabbit Osteochondral Defect Model

Efficacy evaluation of PD sheets in rabbits was performed as described previously [12]. A total of 69 female Japanese white rabbits (average weight = 3.0 kg: Tokyo Laboratory Animals Science Co., Tokyo, Japan) was used. Before surgery, the rabbits were randomly assigned by weight to either the defect-only group or to PD sheet transplantation groups. For immunosuppression, tacrolimus (1.6 mg/kg/day) was administered intramuscularly daily for 10 days, starting two days before transplantation and then every other day until four weeks after surgery. The rabbits were anesthetized with 2 L/min nitrous oxide, 1 L/min oxygen, and 2.5–3.0% isoflurane (Pfizer, New York, NY, USA) before surgery and transplantation. An osteochondral defect (diameter = 5 mm; depth = 3 mm) was created in the patellar groove of the femur using a 5-mm biopsy punch (Kai Industries, Gifu, Japan) as a marking guide and a 5-mm drill. Slight bleeding from the subchondral bone was confirmed, and physiological saline (Nipro, Osaka, Japan) was used to clean the defect and to prevent thermal damage.

#### 4.3.2. PD Sheet Transplantation

For PD sheet transplantation, the culture plates containing the PD sheets were kept at 25 °C for 30 min to allow their detachment. One PD sheet per knee was transplanted on the osteochondral defect using a polyvinylidene difluoride membrane. After restoration of the patella, the quadriceps femoris muscle and tendon were sutured to prevent dislocation.

#### 4.3.3. Histological Scoring for Regenerated Cartilage

Four weeks after PD sheet transplantation, the rabbits were euthanized by an intravenous administration of 50 mg/mL pentobarbital (Tokyo Chemical Industry, Tokyo, Japan). The operated-on femur was collected and fixed in 20% formalin (Wako Pure Chemical Industries) for 3–5 days. The specimen was decalcified in 10% EDTA (Wako Pure Chemical Industries) for 3–4 weeks and paraffin embedded. The serial sections (3 μm) were cut near the center of the defect area, parallel to the long axis of the femur. For histological examination, sections were stained with hematoxylin and eosin (H&E), safranin-O, and fast green according to standard protocols. The stained sections were randomized and scored separately by two trained orthopedic surgeons, using a modified version of the O’Driscoll score and the International cartilage repair society (ICRS) score [47,48] (Details of the scoring method are shown in Supplementary Table S6).

#### 4.3.4. Immunostaining

For type I collagen (COL1) and type II collagen (COL2) immunostaining, deparaffinized 3-μm sections were treated with 0.4% pepsin (Agilent Technologies, Santa Clara, CA, USA) for 30 min at 37 °C for antigen retrieval. The sections were then incubated with 0.4% pepsin (DAKO, Glostrup, Denmark) at 37 °C for 30 min and washed in distilled water, followed by incubation in 0.3% hydrogen peroxide/methanol solution for 30 min at room temperature (RT) to block endogenous peroxidase activity. Next, the sections were washed in PBS, blocked with 2.5% normal goat serum (NGS) for 10 min at RT, and incubated for 3 h at RT with mouse monoclonal antibody in either human COL1 or human COL2 (Kyowa Pharma Chemical Co., Toyama, Japan) diluted at 1:100 with 1% bovine serum albumin (Sigma-Aldrich) in PBS. Finally, the stained sections were washed in PBS, followed by incubation with the ImmPRESS Reagent Anti Mouse Ig (Vector Laboratories, Burlingame, CA, USA) at RT, immersed for 2–8 min in Tris–HCl buffer (pH 7.6) containing 0.02% diaminobenzidine and 0.005% hydrogen peroxide, and then counterstained with H&E.

For human vimentin detection, deparaffinized sections were treated with 10-mM sodium citrate buffer (pH 6.0) for 10 min at 98 °C in a microwave for antigen retrieval. The sections were incubated with 5% NGS containing PBS for blocking, followed by incubation with Alexa Fluor 647-conjugated rabbit monoclonal antibody (Cell Signaling Technology, Danvers, MA, USA) diluted at 1:100 with 1% bovine serum albumin in PBS overnight at 4 °C to detect human vimentin. The sections were washed in distilled water and then mounted and cured with 4′,6-diamidino-2-phenylindole (Vector Laboratories) according to the manufacturer’s instructions. All microscopic images were obtained using a BZ-9000 Generation II fluorescence microscope (Keyence Corp., Osaka, Japan).

### 4.4. Gene Expression Analysis of PD Sheets

#### 4.4.1. RNA Isolation

The PD sheets were disrupted in TRIzol Reagent (Life Technologies, Carlsbad, CA, USA) using SHAKE Master Neo (Bio Medical Science, Japan), and the total RNA was further purified using the Qiagen RNeasy Mini Kit (QIAGEN, Valencia, CA, USA) according to the manufacturer’s instructions. RNA quantity and quality were determined using a Nanodrop One spectrophotometer (Thermo Fisher Scientific Inc.) and an Agilent Bioanalyzer (Agilent Technologies), as recommended.

#### 4.4.2. cRNA Amplification and Labeling

Total RNA was amplified and labeled with cyanine 3 (Cy3) using the Agilent Low Input Quick Amp Labeling Kit, one-color (Agilent Technologies) following the manufacturer’s instructions. Briefly, the total RNA was reversed transcribed to double-strand cDNA using a poly dT-T7 promoter primer. The primer, template RNA, and quality-control transcripts of known concentration and quality were first denatured at 65 °C for 10 min and incubated for 2 h at 40 °C with 5× First-Strand Buffer, 0.1 M dithiothreitol, 10 mM deoxyribonucleotide triphosphate mix, and AffinityScript RNase Block Mix. The AffinityScript enzyme was inactivated at 70 °C for 15 min. cDNA products were then used as templates for in vitro transcription to generate fluorescent cRNA. The cDNA products were mixed with a transcription master mix in the presence of T7 RNA polymerase and Cy3-labeled cytidine triphosphate and incubated at 40 °C for 2 h. Labeled cRNAs were purified using QIAGEN’s RNeasy mini spin columns and eluted in 30 μL of nuclease-free water. After amplification and labeling, the cRNA quantity and degree of cyanine incorporation were determined using a Nanodrop ND-1000 spectrophotometer and an Agilent Bioanalyzer.

#### 4.4.3. Sample Hybridization

For each hybridization, 0.60 μg of Cy3-labeled cRNA were fragmented and hybridized at 65 °C for 17 h using an Agilent SurePrint G3 Human GE v3 8 × 60K Microarray (Design ID: 072363). After washing, the microarrays were scanned using an Agilent DNA microarray scanner.

#### 4.4.4. Identification of Efficacy Correlation Genes

The intensities of each scanned feature were quantified using Agilent feature extraction software version 11.5.1.1, which performs background subtraction. We used only those features that were flagged as error-free (“Detected” flags) and excluded any feature that was flagged as not positive, not significant, not uniform, not above background, saturated, or a population outlier (“Not Detected” and “Compromised” flags). Quantile normalization was performed using Agilent GeneSpring software version 14.9.1. After quantile normalization of the raw signal data, the probes were filtered based on preexisting annotations with a gene symbol and average signal intensity for all samples (higher than 5 coveted log2 intensity). For extraction of the marker genes, Pearson’s correlation coefficients were calculated between the expression level of each of those probes and the individual ICRS scores, then probes with those having more than 0.4 or less than −0.4 were selected. Finally, 443 genes were identified using these criteria with the large variance being more than 0.5 in log2 intensity for all samples. Welch’s *t*-tests were used between the 2 groups that exhibited efficacy and inefficacy: 171 genes were selected with absolute fold change > 2 and corrected *p*-value < 0.05. Hierarchical clustering analysis was performed using Agilent GeneSpring software version 14.9.1. (Similarity Measure: Euclidian, Linkage Rule: Average).

#### 4.4.5. Enrichment Analysis

Efficacy-correlated genes were analyzed using online tools in Metascape (http://metascape.or./) [13]. Functional enrichment was performed in a default setting. Multiple input gene lists containing positively correlated genes and negatively correlated genes were analyzed.

#### 4.4.6. RT-PCR

The total RNA of the PD sheets was converted to cDNA using QuantiTect Reverse Transcription Kit (Qiagen). The TaqMan PreAmp Master Mix Kit (Applied Biosystems, Waltham, MA, USA) was used to preamplify the cDNA. TaqMan Gene Expression Assays (Applied Biosystems) (see Supplementary Table S6) including fluorescent probes and forward/reverse primers were diluted with Tris and EDTA (TE) buffer (1×) according to the manufacturer’s instructions to obtain a final concentration of 0.2× pooled assay mix. The reactions were performed at 50 μL and prepared as follows: 25 μL of TaqMan PreAmp Master Mix (2×), 12.5 μL of pooled assay mix (0.2×), 1 μL of cDNA sample, and nuclease-free water to adjust to the 50 μL total. The thermal cycler GeneAmp PCR System 9700 (Applied Biosystems) was run at 95 °C for 10 min for denaturing, at 95 °C for 14 15-s cycles, and at 60 °C for 4 min for preamplification PCR. The preamplified cDNA products were diluted with TE buffer (1×) at 1:20 for use as templates for real-time RT-PCR analysis. TaqMan real-time PCR was performed using the 7500 Real-Time PCR System (Applied Biosystems) and the probes in Supplementary Table S7. The PCR reaction mixture comprised 10 μL of TaqMan Gene Expression Master Mix (2×), 1 μL of TaqMan Gene Expression Assay (20×), 5 μL of preamplified cDNA, and nuclease-free water to adjust to the 20 μL total. The thermal cycler 7500 Real-Time PCR System was run at 50 °C for 2 min, 95 °C for 10 min, at 95 °C for 40 15-s cycles, and at 60 °C for 1 min for amplification. The cycle threshold (Ct) values were determined by 7500 Real-Time PCR System software v. 2.0.6 (Thermo Fisher Scientific). The relative expression value of each gene (ΔCt value) was derived against the Ct of the internal control ACTB.

### 4.5. Analysis of Proteins Secreted by PD Sheets

#### 4.5.1. Preparation of the Supernatant of the PD sheets

Each PD sheet was cultured in 3 mL of DMEM/F12 (Gibco) supplemented with 1% fetal bovine serum and 1× antibiotic–antimycotic solution (Gibco) for 72 h. The supernatant was collected, and any debris was removed by centrifugation at 13,000 g for 10 min, divided into aliquots, and stored at −80 °C until required.

#### 4.5.2. Proteomic Analysis Using SOMAscan

The supernatant was analyzed using the SOMAscan assay platform (SomaLogic Inc., Boulder, CO, USA), a multiplexed aptamer-based assay detecting 1129 proteins by slow off-rate modified aptamers (SOMAmers) [14]. The assay uses chemically modified nucleotides to transform a protein signal into a nucleotide signal that can be quantified using relative fluorescence on microarrays. We applied rank-based quantile normalization to the raw signal data. Next, probes were filtered based on signal intensity (higher than 6 coveted log2 intensity) to obtain the average intensity for all samples. For extraction of marker proteins, Pearson’s correlation coefficients were calculated between the protein expression level of each of those probes and the individual ICRS scores, with those having more than 0.4 or less than −0.4 being selected. Finally, 112 genes were identified using these criteria, with the large variance being more than 0.5 in log2 intensity for all samples.

#### 4.5.3. Enzyme-Linked Immunosorbent Assay

The supernatants were defrosted, and the concentrations of proteins were determined using ELISA kits for MIA (Roche), DKK1 (R&D Systems, Minneapolis, MN, USA), ESM1 (abcam, Cambridge, UK), GREM1, and NTN4 (Cloud-Clone Corp., Katy, TX, USA).

### 4.6. Statistical Analysis

Numerical result summaries were expressed as a mean and a standard deviation. Analysis of variance was used to analyze ICRS scores, and Dunnett’s multiple comparison was used for post hoc analysis.

## 5. Conclusions

In this study, we have identified efficacy-associated genes that may contribute to hyaline cartilage regeneration via PD sheet transplantation. The identified characteristics could act as markers to predict the in vivo efficacy of using PD sheets. In addition, if the mechanism by which these factors promote cartilage regeneration is further clarified, this will offer a new approach to OA treatment.

## Figures and Tables

**Table 1:** Enriched gene ontology (GO) terms and genes fall into clusters.

Term	Description	LogP	Log (q-Value)	Symbols
GO:0030198	extracellular matrix organization	−14.18	−9.87	ACAN, COL4A2, COL9A3, HAPLN1, DAG1, FOXC1, HSPG2, CCN1, ITGA6, ITGA3, LAMB3, MELTF, PDGFA, SOX9, TNXB, ITGA10, ADAMTSL2, SPINT2, ABI3BP, GREM1, NTN4, THSD4, SCUBE1, OLFML2A, DMD, GDF5, ADAMTS1, P3H2, COL10A1, MMP17, CAPNS2, CTRB2
GO:0001525	angiogenesis	−9.15	−5.53	COL4A2, CSPG4, DAG1, FGF1, FOXC1, HGF, HOXA3, HSPG2, CCN1, LEPR, MFGE8, NOTCH3, PDGFA, PTGIS, FZD8, APLN, ADGRG1, RAMP1, ESM1, GREM1, HIPK2, RASIP1, ISM1, WNT11, ERRFI1, ADRB2, DOK1, EFNB1, GALNT3, IGFBP2, PTGIR, SOX9, MTSS1, PLCB1, NGEF, RHBDF2, SPRY4
GO:0070848	response to growth factor	−7.29	−4.01	RUNX3, COL4A2, FGF1, FOXC1, GALNT3, GDF10, HGF, CCN1, ITGA3, PENK, SOX5, SOX9, GDF5, APLN, ADAMTSL2, SPINT2, DKK1, BAMBI, GREM1, DKK3, HIPK2, ERRFI1, SPRY4, INHBB
GO:0001503	ossification	−6.64	−3.50	ACP5, ADRB2, FOXC1, GDF10, HGF, CCN1, P2RX7, PENK, SRGN, SOX9, WNT3, WNT11, GDF5, DKK1, GREM1, SMOC1, ACAN, RUNX3, HAPLN1, HOXA3, HOXD10, SOX5, SPNS2, CSPG4, LEPR
GO:0003002	regionalization	−6.33	−2.93	EMX1, EVX1, FGF1, FOXC1, HOXA3, HOXA10, HOXD10, LFNG, WNT3, LHX3, ADGRG1, LHX2, ZEB2, SPRY1, DKK1, GREM1, FOXB1, HIPK2, HES2, GSC, SP8, EFNB1, SHROOM3
GO:0048598	embryonic morphogenesis	−5.72	−2.71	COL4A2, DAG1, FOXC1, HOXA3, HOXD10, CCN1, ITGA3, LAMB3, SOX9, WNT3, WNT11, GDF5, APLN, LHX2, SPINT2, DKK1, HIPK2, SHROOM3, ACAN, ADAMTS1, GREM1, HSPB7, ALPK3, SCUBE1, PDGFA
GO:0035272	exocrine system development	−5.57	−2.58	DAG1, FOXC1, HGF, PDGFA, SOX9, NTN4, FGF1, SPINT2, RASIP1, WNT11, DKK1, GREM1, CYP19A1, HOXA3, WNT3, APLN, DKK3, PLCB1, PRR5L, PEAR1, LHX2
GO:0072006	nephron development	−5.52	−2.55	FGF1, FOXC1, ITGA3, NOTCH3, PDGFA, PODXL, SOX9, WNT11, MTSS1, DAG1, HGF, CCN1, WNT3, LHX2, SPINT2, DKK1, RIPK4, RASIP1, SHROOM3, NTN4, CYP19A1, ITGA6, CRLF1, ADAMTS1, ADAMTSL2, ERRFI1, PGF, SPRY1
GO:2000272	negative regulation of signaling receptor activity	−5.38	−2.19	DKK1, DKK3, ERRFI1, LYNX1, MTRNR2L7, MTRNR2L3, MTRNR2L8, MTRNR2L9, ADRB2, MUC4, SHANK2, GREM1
GO:0043408	regulation of mitogen-activated protein kinase cascade	−5.17	−2.35	ADRB2, CSPG4, DAG1, FGF1, GDF10, HGF, CCN1, INHBB, IRAK2, P2RX7, PDGFA, GDF5, FZD8, DKK1, PLCB1, GREM1, HIPK2, ERRFI1, NDRG2, SPRY4, DMD, RASIP1, PRR5L, CAMK2N2, WNT11
GO:0031128	developmental induction	−5.08	−2.30	FGF1, SOX9, WNT3, DKK1, HIPK2, COL4A2, DAG1, FOXC1, ITGA3, LAMB3, WNT11, APLN, ADRB2, IGFBP2, P2RX7, FZD8, SHANK2, PLCB1, BAMBI, GREM1, DKK3, NDRG2, LHX2, ANK1, HGF, HOXD10, HSPG2, NOTCH3, SOX5, RAMP1, GDF5
GO:0061035	regulation of cartilage development	−4.54	−1.84	CCN1, SOX5, SOX9, WNT11, GDF5, GREM1, ACAN, RUNX3, HOXA3, PDGFA, ANXA2, GDF10, SRGN, DKK1
GO:0072497	mesenchymal stem cell differentiation	−4.52	−1.83	SOX5, SOX9, WNT3, FOXC1, TNXB, THSD4, ADAMTSL2, ERRFI1, DKK1

## Supplementary Materials

Supplementary materials can be found at https://www.mdpi.com/1422-0067/21/1/149/s1.

## Abbreviations

ACAN	Aggrecan
ACTB	Actin beta
APOE	Apolipoprotein E
CLS1	Collagenase Type I
COL1	Type I collagen
COL2	Type II collagen
Ct	Cycle threshold
Cy3	Cyanine 3
ΔCt	Delta Ct
DKK1	Dickkopf WNT signaling pathway inhibitor 1
DMEM/F12	Dulbecco’s modified Eagle’s medium–Nutrient Mixture F-12
EDTA	Ethylenediaminetetraacetic acid
ELISA	Enzyme-linked immunosorbent assay
ESM1	Endothelial cell-specific molecule 1
FABP3	Fatty acid-binding protein 3
GDF5	Growth differentiation factor 5
GES	Gene expression score
GO	Gene ontology
GREM1	Gremlin 1
H&E	Hematoxylin and eosin
ICRS	International Cartilage Repair Society
ITGA10	Integrin alpha 10
MIA	Melanoma inhibitory activity
MSC	Mesenchymal stem cells
NGS	Normal goat serum
NTN4	Netrin 4
OA	Osteoarthritis
PBS	Phosphate buffer saline
PCR	Polymerase chain reaction
PD	Polydactyly-derived
PD sheet	PD chondrocyte sheet
PDGFA	Platelet-derived growth factor subunit A
PGF	Placental growth factor
RARRES2	Retinoic acid receptor responder 2
RT	Room temperature
RT-PCR	Reverse transcription PCR
SERPINA3	Serpin family A member 3
SOMA	Slow off-rate modified aptamers
SOX5	SRY-box transcription factor 5
SOX9	SRY-box transcription factor 9
SRY	Sex-determining region Y
TE	Tris and EDTA

## References

[1] Yoshimura N., Muraki S., Oka H., Mabuchi A., En-Yo Y., Yoshida M., Saika A., Yoshida H., Suzuki T., Yamamoto S. (2009). Prevalence of knee osteoarthritis, lumbar spondylosis, and osteoporosis in Japanese men and women: The research on osteoarthritis/osteoporosis against disability study. J. Bone Miner. Metab..

[2] Okano T., Yamada N., Sakai H., Sakurai Y. (1993). A novel recovery system for cultured cells using plasma-treated polystyrene dishes grafted with poly (N-isopropylacrylamide). J. Biomed. Mater. Res..

[3] Okano T., Yamada N., Okuhara M., Sakai H., Sakurai Y. (1995). Mechanism of cell detachment from temperature-modulated, hydrophilic-hydrophobic polymer surfaces. Biomaterials.

[4] Kaneshiro N., Sato M., Ishihara M., Mitani G., Sakai H., Mochida J. (2006). Bioengineered chondrocyte sheets may be potentially useful for the treatment of partial thickness defects of articular cartilage. Biochem. Biophys. Res. Commun..

[5] Ebihara G., Sato M., Yamato M., Mitani G., Kutsuna T., Nagai T., Ito S., Ukai T., Kobayashi M., Kokubo M. (2012). Cartilage repair in transplanted scaffold-free chondrocyte sheets using a minipig model. Biomaterials.

[6] Ito S., Sato M., Yamato M., Mitani G., Kutsuna T., Nagai T., Ukai T., Kobayashi M., Kokubo M., Okano T. (2012). Repair of articular cartilage defect with layered chondrocyte sheets and cultured synovial cells. Biomaterials.

[7] Tani Y., Sato M., Maehara M., Nagashima H., Yokoyama M., Yokoyama M., Yamato M., Okano T., Mochida J. (2017). The effects of using vitrified chondrocyte sheets on pain alleviation and articular cartilage repair. J. Tissue Eng. Regen. Med..

[8] Takatori N., Sato M., Toyoda E., Takahashi T., Okada E., Maehara M., Watanabe M. (2018). Cartilage repair and inhibition of the progression of cartilage degeneration after transplantation of allogeneic chondrocyte sheets in a nontraumatic early arthritis model. Regen. Ther..

[9] Sato M., Yamato M., Mitani G., Takagaki T., Hamahashi K., Nakamura Y., Ishihara M., Matoba R., Kobayashi H., Okano T. (2019). Combined surgery and chondrocyte cell-sheet transplantation improves clinical and structural outcomes in knee osteoarthritis. NPJ Regen. Med..

[10] Yanke A.B., Tilton A.K., Wetters N.G., Merkow D.B., Cole B.J. (2015). DeNovo NT Particulated Juvenile Cartilage Implant. Sports Med. Arthrosc..

[11] Maehara M., Sato M., Toyoda E., Takahashi T., Okada E., Kotoku T., Watanabe M. (2017). Characterization of polydactyly-derived chondrocyte sheets versus adult chondrocyte sheets for articular cartilage repair. Inflamm. Regen..

[12] Takahashi T., Sato M., Toyoda E., Maehara M., Takizawa D., Maruki H., Tominaga A., Okada E., Okazaki K., Watanabe M. (2018). Rabbit xenogeneic transplantation model for evaluating human chondrocyte sheets used in articular cartilage repair. J. Tissue Eng. Regen. Med..

[13] Zhou Y., Zhou B., Pache L., Chang M., Khodabakhshi A.H., Tanaseichuk O., Benner C., Chanda S.K. (2019). Metascape provides a biologist-oriented resource for the analysis of systems-level datasets. Nat. Commun..

[14] Gold L., Ayers D., Bertino J., Bock C., Bock A., Brody E.N., Carter J., Dalby A.B., Eaton B.E., Fitzwater T. (2010). Aptamer-based multiplexed proteomic technology for biomarker discovery. PLoS ONE.

[15] Gold L., Walker J.J., Wilcox S.K., Williams S. (2012). Advances in human proteomics at high scale with the SOMAscan proteomics platform. New Biotechnol..

[16] Oh H., Chun C.-H., Chun J.-S. (2012). Dkk-1 expression in chondrocytes inhibits experimental osteoarthritic cartilage destruction in mice. Arthr. Rheum..

[17] Leijten J.C.H., Moreira Teixeira L.S., Landman E.B.M., van Blitterswijk C.A., Karperien M. (2012). Hypoxia inhibits hypertrophic differentiation and endochondral ossification in explanted tibiae. PLoS ONE.

[18] Bosserhoff A.K., Buettner R. (2003). Establishing the protein MIA (melanoma inhibitory activity) as a marker for chondrocyte differentiation. Biomaterials.

[19] Schubert T., Schlegel J., Schmid R., Opolka A., Grassel S., Humphries M., Bosserhoff A.-K. (2010). Modulation of cartilage differentiation by melanoma inhibiting activity/cartilage-derived retinoic acid-sensitive protein (MIA/CD-RAP). Exp. Mol. Med..

[20] Tscheudschilsuren G., Bosserhoff A.K., Schlegel J., Vollmer D., Anton A., Alt V., Schnettler R., Brandt J., Proetzel G. (2006). Regulation of mesenchymal stem cell and chondrocyte differentiation by MIA. Exp. Cell Res..

[21] Takaku Y., Murai K., Ukai T., Ito S., Kokubo M., Satoh M., Kobayashi E., Yamato M., Okano T., Takeuchi M. (2014). In vivo cell tracking by bioluminescence imaging after transplantation of bioengineered cell sheets to the knee joint. Biomaterials.

[22] Kaneshiro N., Sato M., Ishihara M., Mitani G., Sakai H., Kikuchi T., Mochida J. (2007). Cultured articular chondrocytes sheets for partial thickness cartilage defects utilizing temperature-responsive culture dishes. Eur. Cell Mater..

[23] Hamahashi K., Sato M., Yamato M., Kokubo M., Mitani G., Ito S., Nagai T., Ebihara G., Kutsuna T., Okano T. (2015). Studies of the humoral factors produced by layered chondrocyte sheets. J. Tissue Eng. Regen. Med..

[24] Kokubo M., Sato M., Yamato M., Mitani G., Kutsuna T., Ebihara G., Okano T., Mochida J. (2016). Characterization of chondrocyte sheets prepared using a co-culture method with temperature-responsive culture inserts. J. Tissue Eng. Regen. Med..

[25] Dell’accio F., De Bari C., Eltawil N.M., Vanhummelen P., Pitzalis C. (2008). Identification of the molecular response of articular cartilage to injury, by microarray screening: Wnt-16 expression and signaling after injury and in osteoarthritis. Arthr. Rheum..

[26] Huang X., Zhong L., van Helvoort E., Lafeber F., Mastbergen S., Hendriks J., Post J.N., Karperien M. (2019). The Expressions of Dickkopf-Related Protein 1 and Frizzled-Related Protein Are Negatively Correlated to Local Inflammation and Osteoarthritis Severity. Cartilage.

[27] Leijten J.C.H., Bos S.D., Landman E.B.M., Georgi N., Jahr H., Meulenbelt I., Post J.N., van Blitterswijk C.A., Karperien M. (2013). GREM1, FRZB and DKK1 mRNA levels correlate with osteoarthritis and are regulated by osteoarthritis-associated factors. Arthr. Res. Ther..

[28] Loughlin J., Dowling B., Chapman K., Marcelline L., Mustafa Z., Southam L., Ferreira A., Ciesielski C., Carson D.A., Corr M. (2004). Functional variants within the secreted frizzled-related protein 3 gene are associated with hip osteoarthritis in females. Proc. Natl. Acad. Sci. USA.

[29] He F., Soriano P. (2017). Dysregulated PDGFRα signaling alters coronal suture morphogenesis and leads to craniosynostosis through endochondral ossification. Development.

[30] Peracchia F., Ferrari G., Poggi A., Rotilio D. (1991). IL-1 beta-induced expression of PDGF-AA isoform in rabbit articular chondrocytes is modulated by TGF-beta 1. Exp. Cell Res..

[31] Yao Z., Chen P., Wang S., Deng G., Hu Y., Lin Q., Zhang X., Yu B. (2019). Reduced PDGF-AA in subchondral bone leads to articular cartilage degeneration after strenuous running. J. Cell. Physiol..

[32] Enoki Y., Sato T., Kokabu S., Hayashi N., Iwata T., Yamato M., Usui M., Matsumoto M., Tomoda T., Ariyoshi W. (2017). Netrin-4 Promotes Differentiation and Migration of Osteoblasts. In Vivo.

[33] Zhou Z., Xie J., Lee D., Liu Y., Jung J., Zhou L., Xiong S., Mei L., Xiong W.-C. (2010). Neogenin regulation of BMP-induced canonical Smad signaling and endochondral bone formation. Dev. Cell.

[34] Han Y., Shao Y., Liu T., Qu Y.-L., Li W., Liu Z. (2015). Therapeutic effects of topical netrin-4 inhibits corneal neovascularization in alkali-burn rats. PLoS ONE.

[35] Lejmi E., Leconte L., Pédron-Mazoyer S., Ropert S., Raoul W., Lavalette S., Bouras I., Feron J.-G., Maitre-Boube M., Assayag F. (2008). Netrin-4 inhibits angiogenesis via binding to neogenin and recruitment of Unc5B. Proc. Natl. Acad. Sci. USA.

[36] Bechard D., Meignin V., Scherpereel A., Oudin S., Kervoaze G., Bertheau P., Janin A., Tonnel A., Lassalle P. (2000). Characterization of the secreted form of endothelial-cell-specific molecule 1 by specific monoclonal antibodies. J. Vasc. Res..

[37] Shin J.W., Huggenberger R., Detmar M. (2008). Transcriptional profiling of VEGF-A and VEGF-C target genes in lymphatic endothelium reveals endothelial-specific molecule-1 as a novel mediator of lymphangiogenesis. Blood.

[38] Mori Y., Chung U., Tanaka S., Saito T. (2014). Determination of differential gene expression profiles in superficial and deeper zones of mature rat articular cartilage using RNA sequencing of laser microdissected tissue specimens. Biomed. Res..

[39] Rocha S.F., Schiller M., Jing D., Li H., Butz S., Vestweber D., Biljes D., Drexler H.C.A., Nieminen-Kelhä M., Vajkoczy P. (2014). Esm1 modulates endothelial tip cell behavior and vascular permeability by enhancing VEGF bioavailability. Circ. Res..

[40] Nagai T., Furukawa K.S., Sato M., Ushida T., Mochida J. (2008). Characteristics of a scaffold-free articular chondrocyte plate grown in rotational culture. Tissue Eng. Part A.

[41] Nagai T., Sato M., Kutsuna T., Kokubo M., Ebihara G., Ohta N., Mochida J. (2010). Intravenous administration of anti-vascular endothelial growth factor humanized monoclonal antibody bevacizumab improves articular cartilage repair. Arthr. Res. Ther..

[42] Boeuf S., Steck E., Pelttari K., Hennig T., Buneb A., Benz K., Witte D., Sültmann H., Poustka A., Richter W. (2008). Subtractive gene expression profiling of articular cartilage and mesenchymal stem cells: Serpins as cartilage-relevant differentiation markers. Osteoarthr. Cartil..

[43] Bensaad K., Favaro E., Lewis C.A., Peck B., Lord S., Collins J.M., Pinnick K.E., Wigfield S., Buffa F.M., Li J.-L. (2014). Fatty acid uptake and lipid storage induced by HIF-1α contribute to cell growth and survival after hypoxia-reoxygenation. Cell Rep..

[44] Wang S., Zhou Y., Andreyev O., Hoyt R.F., Singh A., Hunt T., Horvath K.A. (2014). Overexpression of FABP3 inhibits human bone marrow derived mesenchymal stem cell proliferation but enhances their survival in hypoxia. Exp. Cell Res..

[45] Hunt H.E., Sadr K., Deyoung A.J., Gortz S., Bugbee W.D. (2014). The role of immunologic response in fresh osteochondral allografting of the knee. Am. J. Sports Med..

[46] Ryan P.M., Turner R.C., Anderson C.D., Groth A.T. (2018). Comparative Outcomes for the Treatment of Articular Cartilage Lesions in the Ankle with a DeNovo NT Natural Tissue Graft: Open Versus Arthroscopic Treatment. Orthop. J. Sports Med..

[47] Mainil-Varlet P., Aigner T., Brittberg M., Bullough P., Hollander A., Hunziker E., Kandel R., Nehrer S., Pritzker K., Roberts S. (2003). Histological Assessment of Cartilage Repair. J. Bone Jt. Surg..

[48] O’Driscoll S.W., Keeley F.W., Salter R.B. (1986). The chondrogenic potential of free autogenous periosteal grafts for biological resurfacing of major full-thickness defects in joint surfaces under the influence of continuous passive motion. An experimental investigation in the rabbit. J. Bone Jt. Surg. Am..

