# Supplementary material for: Transcriptomic and Proteomic Analyses Reveal the Potential Mode of Action of Chondrocyte Sheets in Hyaline Cartilage Regeneration
Source: Int J Mol Sci. 2019 Dec 24;21(1):149. doi: 10.3390/ijms21010149 (PMC6981399; doi:10.3390/ijms21010149)
Supplement: Supplementary file 1 [file ijms-21-00149-s001.pdf]

Supplementary Table 1

The details of PD cells used for PD-sheets fabrication

ID	Gender	Months	Plating	Passage	Freezing medium
Stock 1	N.A.	N.A.	explant	N.A.	SCB
Stock 2	F	17	cell	P2	SCB
Stock 3	M	15	cell	P2	SCB
Stock 4	F	15	cell	P6	SCB
Stock 5	N.A.	N.A.	explant	N.A.	TC protector
Stock 6	M	8	cell	P2	SCB
Stock 7	F	10	cell	P2	SCB
Stock 8	F	10	cell	P2	SCB
Stock 9	F	23	cell	P2	SCB
Stock 10	F	15	cell	P3	SCB
Stock 11	M	12	cell	P2	SCB
Stock 12	M	12	cell	P4	SCB

Stock4 and Stock10 derived from same donor.

Stock7 and Stock8 derived from same donor of different body parts.

All other stocks derived from different donors.

N.A.; data not available, SCB; STEM-CELLBANKER,

Supplementary Table 2

Top20 genes of positive correlation

Gene ID	Gene Symbol	GeneName	Corr
25791	NGEF	neuronal guanine nucleotide exchange factor	0.81
388115	C15orf52	chromosome 15 open reading frame 52	0.80
3200	HOXA3	homeobox A3	0.80
3914	LAMB3	laminin, beta 3	0.80
375719	AQP7P1	aquaporin 7 pseudogene 1	0.77
27122	DKK3	dickkopf WNT signaling pathway inhibitor 3	0.76
864	RUNX3	runt-related transcription factor 3	0.72
6662	SOX9	SRY (sex determining region Y)-box 9	0.71
1605	DAG1	dystroglycan 1 (dystrophin-associated glycoprotein 1)	0.70
91683	SYT12	synaptotagmin XII	0.70
51655	RASD1	RAS, dexamethasone-induced 1	0.69
57538	ALPK3	alpha-kinase 3	0.69
3655	ITGA6	integrin, alpha 6	0.69
9510	ADAMTS1	ADAM metallopeptidase with thrombospondin type 1 motif, 1	0.68
28996	HIPK2	homeodomain interacting protein kinase 2	0.67
54206	ERRFI1	ERBB receptor feedback inhibitor 1	0.67
9788	MTSS1	metastasis suppressor 1	0.67
6649	SOD3	superoxide dismutase 3, extracellular	0.66
317762	CCDC85C	coiled-coil domain containing 85C	0.65
4241	MFI2	antigen p97 (melanoma associated) identified by monoclonal antibodies 133.2 and 96.5	0.64

Supplementary Table 3　Top20 genes of negative correlation

Gene ID	Gene Symbol	GeneName	Corr
65980	BRD9	bromodomain containing 9	-0.81
286319	TUSC1	tumor suppressor candidate 1	-0.81
10252	SPRY1	sprouty homolog 1, antagonist of FGF signaling (Drosophila)	-0.80
388759	C1orf229	chromosome 1 open reading frame 229	-0.80
84264	HAGHL	hydroxyacylglutathione hydrolase-like	-0.80
400604	TOB1-AS1	TOB1 antisense RNA 1	-0.80
27023	FOXB1	forkhead box B1	-0.79
113091	PTH2	parathyroid hormone 2	-0.79
218	ALDH3A1	aldehyde dehydrogenase 3 family, member A1	-0.79
55731	FAM222B	family with sequence similarity 222, member B	-0.78
256933	NPB	neuropeptide B	-0.78
85294	KRTAP2-4	keratin associated protein 2-4	-0.77
284365	MGC45922	uncharacterized LOC284365	-0.77
102724362	LOC102724362	uncharacterized LOC102724362	-0.77
7755	ZNF205	zinc finger protein 205	-0.77
80164	PRR36	proline rich 36	-0.77
27068	PPA2	pyrophosphatase (inorganic) 2	-0.77
94097	SFXN5	sideroflexin 5	-0.77
92597	MOB1B	MOB kinase activator 1B	-0.76
101928760	LOC101928760	uncharacterized LOC101928760	-0.76
100287098	LOC100287098	uncharacterized LOC100287098	-0.76

Supplementary Table 4　Top20 secreted proteins of positive correlation

Gene ID	Symbol	Description	r
81035	COLEC12	collectin subfamily member 12	0.7
1520	CTSS	cathepsin S	0.68
11082	ESM1	endothelial cell specific molecule 1	0.64
2512	FTL	ferritin light chain	0.61
2495	FTH1	ferritin heavy chain 1	0.61
26585	GREM1	gremlin 1, DAN family BMP antagonist	0.59
2896	GRN	granulin precursor	0.56
12	SERPINA3	serpin family A member 3	0.56
54205	CYCS	cytochrome c, somatic	0.54
3570	IL6R	interleukin 6 receptor	0.52
22943	DKK1	dickkopf WNT signaling pathway inhibitor 1	0.52
7058	THBS2	thrombospondin 2	0.51
5580	PRKCD	protein kinase C delta	0.51
6590	SLPI	secretory leukocyte peptidase inhibitor	0.5
3593	IL12B	interleukin 12B	0.48
51561	IL23A	interleukin 23 subunit alpha	0.48
9902	MRC2	mannose receptor C type 2	0.48
27121	DKK4	dickkopf WNT signaling pathway inhibitor 4	0.48
51506	UFC1	ubiquitin-fold modifier conjugating enzyme 1	0.47
6750	SST	somatostatin	0.47

Supplementary Table 5　Top20 secreted proteins of negative correlation

Gene ID	Symbol	Description	r
4666	NACA	nascent polypeptide-associated complex alpha subunit	-0.75
6372	CXCL6	C-X-C motif chemokine ligand 6	-0.75
10924	SMPDL3A	sphingomyelin phosphodiesterase acid like 3A	-0.71
27120	DKKL1	dickkopf like acrosomal protein 1	-0.7
5617	PRL	prolactin	-0.7
7006	TEC	tec protein tyrosine kinase	-0.68
6356	CCL11	C-C motif chemokine ligand 11	-0.67
3553	IL1B	interleukin 1 beta	-0.65
3459	IFNGR1	interferon gamma receptor 1	-0.62
6387	CXCL12	C-X-C motif chemokine ligand 12	-0.62
6885	MAP3K7	mitogen-activated protein kinase kinase kinase 7	-0.61
10454	TAB1	TGF-beta activated kinase 1 (MAP3K7) binding protein 1	-0.61
4982	TNFRSF11B	TNF receptor superfamily member 11b	-0.6
7076	TIMP1	TIMP metallopeptidase inhibitor 1	-0.6
1933	EEF1B2	eukaryotic translation elongation factor 1 beta 2	-0.59
2050	EPHB4	EPH receptor B4	-0.58
3791	KDR	kinase insert domain receptor	-0.58
51129	ANGPTL4	angiopoietin like 4	-0.58
8576	STK16	serine/threonine kinase 16	-0.56
8742	TNFSF12	TNF superfamily member 12	-0.55

Supplementary Table 6 . ICRS histological grading system

Item	Description
Ti: Tissue morphology	
	1: Exclusively not cartilage
	2: Mostly not cartilage
	3: Mostly fibrocartilage
	4: Mostly hyaline cartilage
Matx: Matrix staining	
	1: None
	2: Slight
	3: Moderate
	4: Strong
Stru: Structural integrity	
	1: Severe disintegration
	2: Cysts or disruptions
	3: No organization of chondrocytes
	4: Beginning of columnar organization of chondrocytes
	5: Normal, similar to healthy mature cartilage
Clus: Chondrocyte clustering in implant	
	1: 25–100% of the cells clustered
	2: < 25% of the cells clustered
	3: No clusters
Tide: Intactness of the calcified cartilage layer, formation of tidemark	
	1: < 25% of the calcified cartilage layer intact
	2: 25–49% of the calcified cartilage layer intact
	3: 50–75% of the calcified cartilage layer intact
	4: 76–90% of the calcified cartilage layer intact
	5: Completely intact calcified cartilage layer
Bform: Subchondral bone formation	
	1: No formation
	2: Slight
	3: Strong
SurfH: Histological appraisal of surface architecture	
	1: Severe fibrillation or disruption
	2: Moderate fibrillation or irregularity
	3: Slight fibrillation or irregularity

	4: Normal
FilH: Histological appraisal defect filling	
	1: < 25%
	2: 26–50%
	3: 51–75%
	4: 76–90%
	5: 91–110%
Latl: Lateral integration of implanted material	
	1: Not bonded
	2: Bonded at one end/partially both ends
	3: Bonded at both sides
Basl: Basal integration of implanted material	
	1: < 50%
	2: 50–70%
	3: 70–90%
	4: 91–100%
InfH: Inflammation	
	1: Strong inflammation
	3: Slight inflammation
	5: No inflammation
**Maximum total**	**45 points**

This system evaluates repaired tissue based on 11 items: tissue morphology (Ti); matrix staining (Matx); structural integrity (Stru); cluster formation (Clus); tidemark opening (Tide); bone formation (Bform); histological appraisal of surface architecture (SurfH); histological appraisal of the degree of defect filling (FilH); lateral integration of defect - filling tissue (Latl); basal integration of defect - filling tissue (Basl); and histological signs of inflammation (InfH). The total score range is 11–45.

Supplementary Table 7 Probes used in TaqMAN gene expression assay

Gene Symbol	Assay ID	Reference Sequence
ACAN	Hs00153936_m1	NM_013227.3; NM_001135.3
ACTB	Hs01060665_g1	NM_001101.3
GDF5	Hs00167060_m1	NM_000557.2
ITGA10	Hs01006923_g1	NM_003637.3
SOX9	Hs01001343_g1	NM_000346.3
SOX5	Hs00753050_s1	NM_001261414.1; NM_006940.4; NM_001261415.1; NM_178010.2; NM_152989.3
